# The family talk intervention in palliative care: a study protocol

**DOI:** 10.1186/s12904-018-0290-8

**Published:** 2018-02-23

**Authors:** Rakel Eklund, Ulrika Kreicbergs, Anette Alvariza, Malin Lövgren

**Affiliations:** 1 Department of Health Care Sciences, Palliative Research Centre, Ersta Sköndal Bräcke University College, Box 11189, 100 61 Stockholm, Sweden; 2 The Department of Women’s and Children’s Health, Paediatric Oncology and Haematology, Karolinska Institutet, Karolinska University Hospital, Astrid Lindgren Children’s Hospital, Childhood Cancer Research Unit, 177 77 Stockholm, Sweden; 3 Capio Palliative Care, Dalen Hospital, 121 87 Stockholm, Sweden

**Keywords:** Palliative care, Family, Minor children, Complex intervention, Study protocol, Family talk intervention

## Abstract

**Background:**

In palliative care contexts, support programs for families with a severely ill parent and minor children are few, and even fewer have been evaluated scientifically. The aims of this study are to examine feasibility and potential effects of a modified version of the Family Talk Intervention (FTI) in palliative care.

**Methods:**

This ongoing family-centered intervention has a quasi-experimental design comparing one intervention and one comparison group. The intervention includes severely ill parents who have minor children (aged 6–19 yrs) and are receiving advanced homecare in Stockholm, Sweden between March 2017 and March 2018. The main goal of the FTI is to support family communication through psycho-education and narrative theory. The modified FTI consists of six meetings with family members, and is held by two interventionists. Each family sets up needs-based goals for the intervention. For evaluation purposes, data are collected by questionnaire before the intervention, within two months after baseline, and one year after baseline. Interviews will be conducted within two months after FTI is completed. Notes taken by one of the interventionists during the family meetings will also be used. Questionnaire data analysis will focus on patterns over time using descriptive statistics. For interview data and notes, content analysis will be used.

**Discussion:**

This study will add knowledge about palliative care for parents who have minor children. It will contribute by testing use of FTI in palliative care, and point out directions for future evaluations of FTI in palliative care settings.

**Trial registration:**

ClinicalTrials.gov Identifier NCT03119545, retrospectively registered in April 18, 2017.

## Background

There are few evaluated interventions that focus on families where a parent of minor children is being cared for in palliative care [1, 2]. Of these interventions, most are therapeutic with focus on grief [2]. The present study is the first to evaluate a modified version of the Family Talk Intervention (FTI) in specialized palliative homecare in Sweden and directly targeting families where a parent with minor children is severely ill.

FTI initially derives from the psychiatric context and targets families where a parent has an affective disorder. The intervention is family-centered and aims to help the family develop new perspectives on the illness, communicate among themselves, and to support the parent-child relationship [3, 4]. Modified forms of FTI have been evaluated in the context of somatic disease, and shown to alleviate psychiatric symptoms among parents [5]. Parents also described gaining insight into their children’s thoughts and reactions, reduced conflicts and increased family communication [6]. The children described that the intervention helped them feel more secure, increased their knowledge about the illness, and lessened their negative feelings [7].

Guilt and fear of having caused the parent’s illness is common among children and adolescents living with a parent who is severely ill. These feelings may arise because of insufficient or unsuitable information for children. When children do not receive enough information regarding their parent’s illness or death, they often fantasize to fill in the gaps. These fantasies are not always realistic and could exacerbate the child’s fear or worry [7–9].

Children and adolescents with seriously ill and dying parents have an increased risk for mental illness, e.g. long-term depression [10], anxiety [10, 11], sleeping problems, behavioral issues and problems in school [12]. Living with severe illness and also having minor children, naturally affects parenthood. Parents, just like their children, experience guilt, for instance because of lack of energy and time to be with their children. These parents also have a lot of thoughts about how their illness affects their children and wonder about the short- and long-term impact the imminent death might have on them [13, 14].

## Methods/design

### Aims

The aims of this study are to examine the feasibility of using a modified version of the FTI in palliative care (Aim 1) and to explore potential intervention effects on family communication, knowledge about the illness and psychosocial well-being among participating family members (Aim 2).

### Study design

This is a complex intervention study with several interacting components, in a specific setting and tailored to fit these circumstances [15], namely among families where a parent with minor children is severely ill and needs palliative care. The study has a quasi-experimental design, with non-randomized subject allocation. It involves an intervention group that will have the modified FTI and a comparison group that will receive standard care. Mixed-method will be used.

### A modified version of the family talk intervention

FTI is not a psychotherapeutic intervention; rather, the goal is to open up to honest and family-centered communication and is based on psycho-education and narrative theory [3, 4, 16, 17]. The original intervention entails up to 11 meetings [4], but for this study, FTI has been slightly modified, in close cooperation with two interventionists with experience of working with FTI in clinical practice in palliative care. To adjust for circumstances in the palliative context, e.g. shortage of time for the family and a challenging situation for all family members, the intervention is shortened to six meetings (Table 1), and two interventionists work in pairs with the family. The interventionists follow a manual that covers the content of the FTI. In this manual (called the logbook) the interventionists take notes from every meeting. Each meeting lasts 1–2 h and is held once a week at a place chosen by the family. The two interventionists are trained to conduct the FTI, and also have extensive experience of working with FTI in palliative care among families where a parent is severely ill. The modified version of FTI has been found manageable and satisfactory in clinical practice for families and interventionists in this context but it has not previously been evaluated scientifically.

**Table 1:** The Family Talk Intervention: the focus at each meeting and the family members involved

Meeting 1–6	Involved family members	Focus at the meeting
Meeting 1	Parents	The ill parent’s history.
		To set up the family’s goals for the intervention.
Meeting 2	Parents	The well parent’s history.
Meeting 3	Each child (preferably without the parents)	The child’s understanding of the illness and the situation, worries and questions
Meeting 4	Parents	Summary about worries and questions from meeting 3.
		Planning the family talk (meeting 5).
Meeting 5	Parents and children	“The family talk”. Preferably led by the parents and consisting of questions from both children and parents.
Meeting 6	Parents and sometimes children	Follow-up with focus on how to communicate within the family in the future to reach the family’s goals.

### Inclusion criteria

For a family to participate in this study, at least one parent needs to be severely ill with palliative care needs, and receiving specialized homecare. The family must also include at least one child aged 6–19 years, but all children in the family are welcome to participate. The family members must speak and understand Swedish. The ill parent defines his/her own family. The family can therefore include partners, grandparents, friends, step-children, ex-partners, or others defined by the ill parent.

### Settings and participants

Recruitment of families is ongoing since March 2017 and will continue until March 2018. Families are recruited through four specialized homecare units in the Stockholm region, all of which have multidisciplinary teams and offer 24-h services. Two units recruit families for the FTI and two units recruit families for the comparison group. All families that meet the inclusion criteria are identified by a social worker at each unit. Families in the intervention group are contacted by phone by the interventionists to set up a meeting where the families are given both oral and written information. Families in the comparison group receive an information letter by post, and one week later they are contacted by phone by one of the researchers.

### Sample size

We expect a sample size of 30 families, based on discussions with the four home care units, about how many patients with minor children that have been enrolled previously at the units. No power is calculated to estimate sample size because it is a small-scaled study and designed as a pilot with no aim to do any hypothesis testing [18, 19].

### Measurements

Feasibility and potential effects are measured by study-specific questionnaires at three time-points. Baseline is placed before intervention start (intervention group) or upon enrollment to advanced homecare (comparison group). The first follow-up is within two months after intervention completion (intervention group) or two months after baseline (comparison group), and the second follow up is one year after baseline. Interviews are held within two months after intervention completion (intervention group). Both the questionnaires and the interviews will focus on the family members’ experiences of participation in the FTI. Collaboration with the contact persons at the units and the interventionists’ experiences of feasibility will be evaluated by observations and interviews.

#### Primary outcome

Self-reported family communication will be measured using study-specific questionnaires and interviews.

#### Secondary outcomes

Self-reported knowledge about the illness and self-reported psychosocial well-being (psychosocial well-being for children and anxiety for adults) will be measured using study-specific questionnaires and interviews.

#### Questionnaires

To capture each family member’s experiences, we developed five versions of the questionnaires (children 6–7 years old; children 8–12 years; adolescents 13–19 years; well parent/adult; ill parent) (Table 2). The questions involve background characteristics, knowledge and information about the illness, communication within the family, and psychosocial well-being. Participants in FTI answer questions with focus on feasibility. Specific questions were also developed for the bereaved.

**Table 2:** The content of the questionnaires and the different time points

	Children, 6–7 years			Children, 8–12 years			Adolescents 13–19 years			Well adult/partner			Ill parent		
	B	F1	F2	B	F1	F2	B	F1	F2	B	F1	F2	B	F1	F2
*Study specific questions:*															
-Background characteristics	X			X			X			X			X		
-Knowledge about the illness	X	X	X	X	X	X	X	X	X	X	X	X	X	X	X
-Communication within the family	X	X	X	X	X	X	X	X	X	X	X	X	X	X	X
*Standardized questionnaires:*															
-Psychosocial well-being (PedsQL)	X	X	X	X	X	X	X	X	X						
-Anxiety (GAD-7)										X	X	X	X	X	X
If intervention:															
*Study-specific questions:*															
-Expectations and goals with participation				X			X			X			X		
-Feasibility					X			X			X			X	
If bereaved:															
*Study-specific questions:*															
-The parent’s/partner’s death		X			X			X			X				
*Standardized questionnaires:*															
-Grief (PG-13)			X			X			X			X			

^B^Baseline. ^F1^Follow-up 1. ^F2^Follow-up 2

##### Questions about family communication and knowledge about the illness

Due to lack of validated instruments in Swedish that measure family communication and knowledge about illness, we constructed study-specific questions according to the method presented by Charlton [20]. We used questions from earlier nationwide surveys about parents or siblings who have lost a child/brother or sister to cancer [21, 22], and teenagers who have lost a parent to cancer [23]. We also used questions from earlier intervention project [24, 25] for our study.

##### Questions about psychosocial well-being

Anxiety in adult participants will be measured at all three time-points using the Generalized Anxiety Disorder (GAD-7) scale [26]. For the children, the Pediatric Quality of Life Inventory version 4.0 (PedsQL) [27, 28] will be used all three times. Grief among bereaved family members (both children and adults) will be measured one year after baseline with the Prolonged Grief Disorder (PG-13) instrument [29].

##### Questions about study participation and feasibility

At baseline, all family members in the intervention group (except the youngest age group) will be asked about their expectations and goals for participating in the FTI. At the first follow up, all family members, except the youngest, will receive questions about the meeting they participated in, how they perceived sharing their thoughts and feelings, if relations within the family changed and if the FTI matched their expectations.

##### Pre-test and pilot of the study-specific questions

The questionnaires were pre-tested and then sent out to families for validation. The questions were validated with eleven children, seven adolescents, and five adults [20]. If the children did not know any person with severe illness they were asked to think about someone they knew, who either was or had been ill. Parents were asked to observe how long it took for the younger children (aged 6–12) to fill in the questionnaires and also report to the research group if any questions had been difficult for the child to understand or answer. Based on the feedback obtained through this validation process, a new questionnaire was developed for children 6–7 years, as some of these children could not differentiate between the response alternatives. We therefore modified them for this age group based on Pediatric Quality of Life Inventory 4.0 [27, 28], using only three response alternatives, represented by happy, neutral and sad smiley faces. Children over the age of seven were able to handle the four response alternatives that we initially used.

#### Interviews

To examine the feasibility and potential effects of the modified version of the FTI, interviews will be conducted with the families after they have participated in the FTI. The families can choose how and where the interviews are conducted and if they want to do the interview as a group or alone. Focus for the interviews’ is on the structure and content of the FTI and what the intervention has meant for each family member. The interviews go through every meeting and what has been positive and negative regarding the structure and content, as well as other experiences throughout the FTI (Aim 1). The family members are also asked about family communication, knowledge about the illness, and psychosocial well-being (Aim 2). In addition, interviews will be conducted with the interventionists and contact persons at the units, to evaluate feasibility from their perspective (Aim 1). All interviews will be recorded and transcribed verbatim.

#### Observations

During the process the following components will be observed: 1) the families’ willingness to participate, 2) dropouts, and 3) missing data (forms and item). Collaborations with the contact persons at the four homecare units will be evaluated on the basis of field notes made by the researchers during the study process. The interventionists’ experiences of feasibility and their fidelity to the modified version of FTI will be observed through examination of the logbooks in which they take notes at every meeting. Examples of information that will be available in the logbook are the families’ goals for FTI, the content of the meetings, which family members participated in each meeting, and special events during the meetings.

#### Data analysis

Data from the questionnaires will be analyzed descriptively with focus on communication, knowledge about the illness and psychosocial well-being within the intervention group and between intervention and comparison group (Aim 2). Each different age group/family member will be analyzed separately. As the sample size will be relatively small, all analyses based on questionnaire data will focus on patterns over time using descriptive statistics. Interview data, answers from the open-ended questions in the questionnaires, field notes, and notes from the logbook will be analyzed with content analysis [30], with focus on the feasibility (Aim 1) and the reported experiences of family communication, knowledge about the illness and psychosocial well-being (Aim 2). Quantitative and qualitative data will be interwoven in order to capture different aspects of the outcomes [31].

## Discussion

This will be the first evaluation of the modified version of the FTI for use in palliative care. It will add knowledge regarding family-centered interventions in palliative care – a topic on which research is currently scarce, especially where children are concerned [1]. A variety of stakeholders, including interventionists, caregivers and families, have been involved in developing the modified version of FTI, which can increase the relevancy of the research [32]. This unique study will be valuable for development of interventions for families with minor children in palliative care. Yet, it touches on several challenging issues that can impact the recruitment of families, data collection, and data analysis.

One challenge to recruitment might be gate-keeping, as clinicians are known to predict that participation in a study might burden the patients and their families [33]. Another recruitment challenge might be that only families that already communicate well would want to participate in this kind of intervention. Kissane, Bloch [34] have created a typology of families in grief, based on family function. They conclude that families that agree to participate in such interventions are well-functioning “supportive” or “conflict-resolving” families; in other words, it is families with high cohesiveness and few conflicts who choose to participate. Whether this will be the case in the present study is unknown, but if so, this could influence the study results by giving a more positive impression of both feasibility and outcomes.

Poor retention of participants is another a risk when studying vulnerable groups [35]. The ill parents’ deteriorating health and the severity of the illness could cause problems in the proposed study, with drop-outs, missing forms and missing data as a result. Retaining the other family members is also a challenge, as they might feel preoccupied in their overwhelming situation, and might not have the energy to participate in interviews or fill out the questionnaires at the three different time-points.

Interpreting the outcomes of this study may be hampered by the many confounding factors involved, such as different family constellations, a wide spread of age of children (6–19 years), differences in diagnosis, illness progression and variations in health status and psychosocial well-being of all family members before diagnosis. However, the use of mixed methods could be seen as a strength, as it gives a deeper understanding of the evaluation, the process, outcomes and potential barriers [36, 37]. Quantitative and qualitative data will complement each other and give a greater understanding of the feasibility of the modified version of FTI in palliative care and in studying the potential effects on family communication, knowledge about the illness and the psychosocial well-being among family members.

Children’s voices are often heard only at second hand, through parental proxy reports, even though studies show poor agreement between parents’ and children’s reports [38]. Approaches that include children as subjects, rather than objects, is becoming increasingly common in research involving children [39]. Few studies have been conducted based on children’s own voices during the process of having a parent with severe illness. This study intends to capture the children’s own voices through questionnaires and interviews, as their experiences are an important part of the whole family experience.

This first evaluation of use of the modified version of FTI in families where a severely ill parent with minor children is receiving palliative care, will help to reduce the knowledge gap concerning interventions for these families. The results may also provide important information regarding feasibility and to what degree it is possible to involve all family members at a time in life when most things are put to an end. The proposed study will hopefully point out directions for further evaluations of FTI in palliative care, and for families in these kinds of situations.

## Abbreviations

FTI: The Family Talk Intervention
GAD-7: Generalized Anxiety Disorder scale
PedsQL: the Pediatric Quality of Life Inventory scale version 4.0
PG-13: Prolonged Grief Disorder instrument
